# Vaccination against COVID-19 in Europe: A Typology Based on Cluster Analysis

**DOI:** 10.3390/ijerph19148603

**Published:** 2022-07-14

**Authors:** Darie Cristea, Irina Zamfirache, Raisa-Gabriela Zamfirescu

**Affiliations:** Faculty of Sociology and Social Work, University of Bucharest, 010181 Bucharest, Romania; irina.zamfirache@unibuc.ro (I.Z.); raisa.zamfirescu@unibuc.ro (R.-G.Z.)

**Keywords:** vaccine, COVID-19, cluster k-mean analysis, vaccine hesitancy, vaccine acceptance, vaccine refusal

## Abstract

This study aims to identify a general typology for the EU27, and subsequently in Romania, regarding the hesitation, acceptance and refusal of vaccination against COVID-19. The analysis we propose below is based on the information contained in Eurobarometer 94.3, the data of which were collected at the beginning of most of the national vaccination campaigns in Europe. Based on the attitudes and opinions expressed by the respondents of the European states (EU27), we constructed with the help of the cluster k-means (SPSS) statistical analysis a typology with four categories on the subject of vaccination against COVID-19. Our study proposes a matrix with five items/scenarios on a scale from total agreement to total disagreement. We chose a typology with four attitudinal types (clusters). We subsequently compared the results of the general European analysis with the cluster typology resulting from the same Eurobarometer, the same set of questions, only for the case of Romania, to see if this analysis sheds a specific light on the fact that Romania had a very low vaccination rate compared to other EU Member States.

## 1. Introduction

Since the beginning of the pandemic and in the context of the implementation of restrictions and limitations at European and national levels, the European Commission has conducted several comprehensive studies focused on the Coronavirus pandemic and its effects. We can mention in this respect the research carried out in August 2020 (EB 93.1/ZA 7649) [1] focusing mainly on the restrictions, limitations and confidence of Europeans in national, regional and European institutions on the capacity to manage the Coronavirus pandemic and its effects. A second study was undertaken in early 2021 (EB 94.3/ZA 7780) [2], with data collection taking place in February and March. This time, along with items on satisfaction with the European Union’s response to the Coronavirus pandemic, acceptance of national and European restrictions and priorities, items with a direct reference to vaccination against COVID-19, were included. The presence of these items is motivated by the fact that the beginning of 2021 brought the start of several national vaccination campaigns against COVID-19, at the date of field data collection, several socio-professional and/or demographic categories being already vaccinated or scheduled for vaccination with one of the three vaccines approved and available at that time in Europe—Pfizer, AstraZeneca and Moderna. A third survey to be mentioned is Eurobarometer 95.1 (ZA 7781) [3] conducted in the spring of 2021, with data collection from the second half of March to mid-April. Compared to the two previous studies, the items included on the topic of the Coronavirus pandemic followed in particular the emotions and financial situation of the respondents in the context of national and European restrictions, but also the activity undertaken on online social networks and the motivation to access them in the last seven days at the time of data collection.

In the spring of 2022, approximately 8.1 million Romanians were vaccinated against COVID-19 with at least one dose, and just over 2.5 million Romanians with booster dose, thus approximately 16.6 million doses of vaccine against COVID-19 were used since the beginning of the vaccination campaign on 27 December 2020, and until now in Romania [4]. In a previously published study also focused on the data contained in the Eurobarometer 94.3 mentioned above, we showed how only 57% of Romanians responded at the time of data collection that they would get vaccinated as soon as possible, placing Romania in 21st place in the EU27 ranking on the intention of the population to be vaccinated against COVID-19 [5]. Thus, based on these figures and Romania’s position on the last places in the European rankings on vaccination intent, we can say that Romania is a negative case of vaccination against COVID-19 compared to the European average.

As in Italy before the pandemic [6], recently, in Romania there has been an increasingly strong discourse against the idea of vaccination, a discourse that we can say reached its peak at the time of the first discussions on a vaccine as a possible remedy in the fight against the Coronavirus pandemic. This increasingly vocal discourse has been interpreted by most as an anti-vaccination movement, but as shown by Yaqub et al. [7], the idea of hesitation should not be confused with the vehement refusal of vaccination. Specifically, *vaccine hesitancy* “is a complex phenomenon, context specific, varies across time, place, and types of vaccine” [6,8]. The limitations, restrictions and high degree of transmissibility of SARS-CoV-2 have led to the need to develop a safe, effective and long-lasting vaccine in the fight against the many new strains of this Coronavirus. Thus, this specific need has become one of the main arguments for the hesitation of vaccination against COVID-19, becoming “a significant barrier to vaccination campaigns” [9] and “a threat to the population’s health globally” [10]. Several countries at the European, and even global, level have encountered difficulties in national vaccination campaigns, difficulties caused by the pro-vaccination public discourse in opposition to the attitudes of the population [11]. Factors that have led to public hesitation over vaccination may vary from area to area [12]: thrombosis and vascular side effects have caused much controversy in Europe [13]; other countries also have a culture of hesitation and refusal to vaccinate [6,14]. In addition to the possible side effects and reluctance embedded in state culture, distrust of state institutions and national government [15] has led to a new dimension of the variety of factors that translates into hesitation about vaccination in general.

The analysis we propose below is based on the information contained in Eurobarometer 94.3, the data of which were collected at the beginning of 2021, *respectively, at the beginning of most of the national vaccination campaigns in Europe*.

The aim of this study is to identify a general typology for EU27 and subsequently one at the Romanian level regarding the hesitation, acceptance and expressed refusal of the respondents regarding the vaccination against COVID-19. Of the three European Commission studies mentioned above, this survey is the most eloquent in showcasing an overview of vaccination against COVID-19.

## 2. Materials and Methods

As mentioned above, the most eloquent Eurobarometer for presenting an overview of the start of the vaccination campaign against COVID-19 in Europe is the one conducted in February–March 2021 (EB 94.3/ZA7780) [2]. Data were collected shortly after European-approved vaccines (Pfizer, AstraZeneca and Moderna) became widely available for several occupational categories, but also in the context of the well-known controversies over immunization with one of the approved vaccines [16,17,18].

The tool applied by the European Commission includes a number of items with a direct reference to vaccination against COVID-19, the respondents’ desire to be immunized, the usefulness of vaccination as the main source in stopping the Coronavirus pandemic, and sources of information in the pandemic. Continuing to focus on European trends, we undertook a cluster analysis of the data available in Eurobarometer 94.3 on vaccination.

Based on the attitudes and opinions expressed by the respondents of the European states (EU27), we constructed with the help of the cluster k-means (SPSS) statistical analysis [19] a typology with four categories on the subject of vaccination against COVID-19 [20] (pp. 45–57). In performing the cluster analysis, we ran several models and came to the conclusion that the 4-cluster typology is the most relevant one when trying to find more complex explanations, which go beyond the simple typology “acceptance/hesitation/refusal”.

The data were collected at the European level between February and March 2021, totaling 28,705 respondents aged 15 and over, the data being collected on the basis of a representative stratified sample and complying with the laws and regulations in force on ethical measures and protection of personal data [20] (pp. 6, 66–67).

We did not include all Eurobarometer respondents in the undertaken cluster analysis, but only those from the 27 Member States (EU27, n = 25,154). Before running the analysis, we recoded the variable on the country of origin creating a single category for Germany, as the data from this type of Eurobarometers are collected with information for East Germany and West Germany, respectively. We also applied a recoding process to the five items included in the k-means cluster analysis, in order to easily track the degree of intensity and subsequently the values obtained, the scale from agreement to disagreement being thus recoded, 1 rendering *total disagreement* and 4 *total agreement*. It should be noted that in order to obtain a national, and even regional, relevance in the analysis undertaken, we worked with weighted data. The k-mean cluster analysis was performed on a matrix with five items/scenarios, for each of which the respondent must declare his attitude on a scale from total agreement to total disagreement (items are presented in Table 1). In order to better observe the nuances and attitudes manifested by the respondents, we opted for a typology with four attitudinal types. With a total of 25,020 respondents (134 non-responses were recorded) from the 27 Member States of the European Union, the balanced distribution of respondents in the cluster should be mentioned, with the difference between the largest and smallest cluster being 3.3% (801 individuals).

We then proceeded to compare the analysis performed at the European general level with the cluster typology resulting from the same Eurobarometer, the same set of questions, only for Romania. We recall that the data we are analyzing were collected in February–March 2021, when the vaccination program was still in its incipient stages in EU countries. By the end of the year, however, Romania is proving to be one of the EU countries with the lowest rates of vaccination against COVID. This comparison could help us to understand this phenomenon.

Based on the answers provided on a scale of 1–4 for each of the five scenarios/items, cluster analysis was expected to identify two opposing and directly contrasting types, namely, respondents convinced of the effectiveness of vaccination and the vaccine against COVID-19 as the main tool to stop the pandemic (*acceptance*) and, on the other hand, those who vehemently oppose the idea of vaccination (*refusal*). Between these two extremities, it was expected that the other two types of hesitation (*vaccine hesitancy*) would remain, these being close from a nuance point of view either rather to acceptance or rather to refusal. However, our analysis of Eurobarometer data shows that the trio of acceptance/hesitation/refusal is much more sophisticated. The three attitudes are based on different reasons and perceptions and, as a result, generate new typologies, which go beyond the simple intention of vaccination.

The resulting typology is an exploratory one. A similar analysis was carried out on the sample from Romania, in order to see what differences appear between the EU27 typology and the one of a European country that proved a great reluctance in front of the vaccination against COVID-19 code.

## 3. Results

### 3.1. Cluster Analysis on a European Level

Table 1 shows the results of the cluster analysis (k-mean). On the first column, the items/scenarios to which the subjects responded can be seen, as mentioned above. It should be noted that some items are positive in terms of vaccination, when it comes to wording, others being negative, so the agreement and disagreement with those statements must be interpreted carefully. Regardless of the orientation of the item in relation to vaccination, we coded the total disagreement with the statement with 1, the partial disagreement with 2, the partial agreement with 3 and the total agreement with 4. This indicates the following reading table of the table—values of 1, 2 will indicate a disagreement with that item. Values 3, 4 indicate agreement with the reference item, while values between 2 and 3, especially those around 2.5, indicate an ambiguous attitude, neither positive nor negative towards the item in question.

**Cluster A** is skeptical of the vaccine against COVID—they believe that the vaccine was developed too quickly to be safe and strongly believes that it will produce unknown side effects.

They are not convinced that the vaccine will stop the pandemic, but this belief is weaker than the two mentioned above. Basically, at the beginning of vaccination, the fear of vaccine was the dominant feature of the anti-vaxxer group, not the fear of vaccine inefficiency (we see that today, a year later, the assessment of the vaccine as ineffective with omicron is a strong narrative in the area of vaccination refusal).

Members of this group are also empathetic to other vaccination skeptics.

**Cluster D** is the most pro-vaccination of the four. As can be seen, the subjects disagree with the idea that the vaccine is not safe and that it will have long-term side effects. However, they accept that it may have side effects rather than being done too quickly to be sure.

It is also the most convinced cluster that the vaccine will stop the pandemic and those who say the most do not understand the reluctance of others to vaccinate.

If group A is a focus on refusal vaccine and D on vaccination acceptance, **clusters B and C** are more complex clusters. Group B has the closest scores to the average of the interval, in almost all indicators—this means that we are talking about hesitation, perhaps insufficient knowledge, misunderstanding of the problem, giving less importance to the pandemic than other clusters, etc. Group **C**, compared to **B**, rather, believes that the vaccine is unsafe and will have unknown long-term side effects. Both clusters (B and C) believe to the same extent that the vaccine will stop the pandemic (C slightly more than B), but by no means as strong as those in group D. Group **C** is less empathetic than **B** in terms of the reluctance of those who do not want to get vaccinated. Basically, group B is a more relaxed group of *skeptics/hesitant*, more tolerant (but not very tolerant) than those in cluster C with other hesitant or those who refuse vaccination, while group C is composed of the *undecided*, somehow *cynical*, perhaps *fatalistic* and quite upset in their assessments of the pandemic. Basically, those in group C associate the vaccine with risks; they do not see it as a very effective solution, but in the end, it is probably the only solution. As an identity element, the lack of empathy with those who refuse the vaccine clearly separates those in C from those in B, as those in C seem very interested in others being vaccinated.

Again, it is useful to remind the time of data collection: February–March 2021.

### 3.2. Cluster Analysis on a Romanian Level

We performed the same type of cluster analysis strictly on the sample for Romania from the Eurobarometer (this type of survey works with samples that are representative of each country) and we obtained four clusters, according to Table 2, with the following characteristics:

**Cluster a**: People who are somewhat afraid of the anti-COVID vaccine because it was developed too quickly, and the side effects it could have long-term; paradoxically, they are somehow convinced that only the vaccine will stop the pandemic and that the EU will make sure that it reaches our country; moreover, such subjects are not at all sympathetic with those who refuse to be vaccinated. Basically, for this category, the vaccine is something that needs to be done, even if it means taking risks. It is a seemingly paradoxical category, but not unusual in Eastern Europe and, somehow, especially in Romania, where, throughout history, the individual has been taught to live with phenomena that he did not always like.

**Cluster b**: They do not think the vaccine was developed too quickly to be safe and they are afraid of long-term side effects to a lesser extent than all the other three groups in the cluster analysis. On the other hand, all the coefficients in this cluster range between 1.95 and 2.58, so somehow they tend to the middle of the scale 1–4, as they match the pattern on any item. Basically, we are talking about a segment of the population that wants to be reasonable against this challenge, does not think that the vaccine is necessarily unsafe, nor that it is a miraculous solution, being the least scared of possible long-term effects, but does not ignore them. Somehow, we see here a mixture of reasonable resignation and a moderate interest in the issue, as opposed to the fatalistic and panic-stricken outlook of group a. Cluster b is moderately related to the reluctance of others to vaccinate. Of course, it is not a group of pro-vaccine, nor anti-vaccine, nor necessarily undecided, but rather what the polls in Romania described as the group of those who will get vaccinated, if necessary, if others who have been vaccinated do not suffer from anything, if in the meantime they find out that they have no other solution.

**Cluster c**: It is clearly a vaccine-oriented cluster, which ticks in this regard all the indicators/items taken into account.

**Cluster d**: People who do not oppose vaccination but have concerns (and probably need information) about the safety, long-term effects, and speed of vaccine development. They are the kind of people who could have been lost to the cause of vaccination if the state failed to alleviate their fears through the information/persuasion campaign it brought. This group is similar to Group C in the European typology described above—but there are two highlighted differences, the fear of unknown side effects being lower in this case than in the previous one, and the tolerance for those who hesitate/do not want to be vaccinated is moderate here, but higher than in group C at the European level.

## 4. Discussion

We have already conducted part of the discussion above, under each cluster table, in order to be able to follow their description more easily. This leaves us to make a comparison between the European cluster scheme and the one corresponding to Romania. It should be noted that Romania is on the second to last place in anti-COVID vaccination in Europe [21].

Both schemes have four clusters. We have named in uppercase (A, B, C, D) the clusters from the EU level and in lowercase those from the level of Romania (a, b, c, d). We did not reorder or rename the clusters, we left them in the order in which they were issued by the SPSS analysis. We will now highlight the differences between them if they are notable.

In short, it was expected that both typologies would have a clear cluster of pro-vaccine and anti-vaccine. Indeed, anti-vaccine clusters exist in both: A and c. The question is whether there are major differences between them. There is only one difference, and it is not so much about the pandemic or vaccination, but about trust in the EU. Romanian anti-vaxxers are much more convinced than European anti-vaxxers in general that the European Union will make sure that the vaccine reaches our country.

The surprise is that, if at the European level we really have a firm pro-vaccination cluster, the items taken into account do not show us at the Romanian level a certain cluster of supporters of vaccination. Although Romanian surveys from the beginning of vaccination (late 2020, early 2021) gave a result between 25 and 30% of the population who would intend to get vaccinated against COVID when the vaccine will be available, taking into account more complex attitudes, such as those highlighted by the battery of items we are referring to, do not visibly identify a homogeneous group of strong supporters of vaccination.

Cluster b in the analysis on Romania corresponds to cluster B at the European level (undecided, moderate interest in the problem, lack of panic). One difference would be that the reasonable and disinterested in Romania believe to a lesser extent than the equivalent European cluster that vaccination is the only way to stop the pandemic, which only connects with the fact that in Romania the hesitation was wider and longer than at the European level.

We also notice an equivalence between types a and C. The scores are high for all items in both clusters: fear of vaccine, belief that there is no better solution to stop the pandemic, belief that there will be enough vaccine for all EU countries, but also a relatively cynical lack of understanding of those who do not want to be vaccinated.

As mentioned, at European level, cluster D is for vaccination advocates: they believe in vaccination, they have little or no fear, they are convinced that the EU will make vaccine available to Member States and do not empathize with those who refuse vaccination. The last cluster left in the series for Romania, cluster d, is not a cluster with a firm orientation, but rather a combination of a and b: I believe in vaccination, they are somehow afraid of the vaccine, but not as panicked as group a and have a more reasonable position, perhaps disinterested in those who refuse vaccination (they neither understand them nor condemn them).

## 5. Conclusions

Of course, it is often easier, in an opinion poll, to ask a direct question, for example, about the intention to vaccinate. Additionally, one can divide the audience into who wants, who does not want, who has not decided, who is waiting to receive more information, etc., depending on the available answers.

Not infrequently, over the last two years we have seen, in opinion polls on the attitude towards vaccination against COVID, a certain dynamic, extremely fast, of these figures, depending on the evolution of the pandemic, restrictions and legal measures, rumors, etc. Not infrequently, we have seen that the real vaccination rates did not confirm the opinion polls. That is why it is useful to group the public on the attitude towards such a problem not only after a direct answer, but also according to indicators that seek to break down the problem to be studied into components that could better explain the subsequent behavior of the public.

Of course, depending on the set of indicators taken into account, we can obtain different descriptions and typologies. However, they all contribute to understanding the backgrounds behind the shift from vaccine attitudes to vaccination behavior, especially in situations that have the ability of imposing themselves quickly and completely on a macrosocial level, such as this pandemic.

Hesitation in the face of vaccination, especially in a shocking and new situation such as the COVID 19 pandemic, is a much more complex phenomenon than mere indecision [14,15,22,23]. Likewise, certain attitudes, acceptance/rejection, appear much more elaborate if instead of a direct question we describe them with specific sets of items.

If we take into account the items provided by the cited Eurobarometer, we can draw the following conclusions regarding the typology of vaccination reporting in the EU and Romania:

- There is only one major difference between the typology built on the same indicators for Romania and that for the entire EU. The EU typology has a vaccination-friendly cluster, an anti-vaccine cluster, one that we have described as undecided and disinterested, and a fatalistic one, dominated by fear of vaccine combined with the belief that there is no other solution to the pandemic.
- The typology for Romania has an anti-vaccine cluster and three nuances of hesitation/indecision. There is no definite pro-vaccine cluster here, which also believes in the effectiveness of the vaccine and its lack of danger. The closest to the description of a pro-vaccination type is the fatalistic cluster (which, as noted, already has an equivalent in the typology found for the EU), in which accepting vaccination as a solution is not exactly a rational and open choice, but rather a small sacrifice on a subjective level.
- To some extent, the fear that the vaccine was developed too soon and the fear of unknown side effects also appear in clusters that accept vaccination as a solution. In practical terms, the real undecided ones are not necessarily fearful, but rather disinterested. Fear of vaccination or lack of confidence in the anti-COVID vaccine does not necessarily explain future behavior regarding vaccination.

## Figures and Tables

**Table 1:** K-mean cluster analysis: attitude types towards vaccination against COVID-19.

*To What Extent Do You Agree or Disagree with Each of the Following Statements?*	Type A	Type B	Type C	Type D
COVID-19 vaccines are being developed, tested and authorized too quickly to be safe	3.58	2.14	3.27	1.54
COVID-19 vaccines could have long term side-effects that we do not know yet	3.68	2.65	3.35	2.17
A vaccine is the only way to end the pandemic	1.92	3.00	3.20	3.75
The European Union is playing a key role in ensuring that we can have access to COVID-19 vaccines in our country	2.65	2.65	3.23	3.25
You do not understand why people are reluctant to get vaccinated	1.42	2.25	3.31	3.66
**% of case in each cluster**	**23.3**	**26.6**	**26.5**	**23.6**
	**5842**	**6643**	**6638**	**5898**

Source: Eurobarometer 94.3/2021, authors’ analysis. n (valid) = 25,020 (EU27).

**Table 2:** K-mean cluster analysis: attitude types towards vaccination against COVID-19.

*To What Extent Do You Agree or Disagree with Each of the Following Statements?*	Type a	Type b	Type c	Type d
COVID-19 vaccines are being developed, tested and authorized too quickly to be safe	3.15	1.95	3.69	3.11
COVID-19 vaccines could have long term side-effects that we do not know yet	3.40	2.39	3.70	2.98
A vaccine is the only way to end the pandemic	3.67	2.37	1.98	3.23
The European Union is playing a key role in ensuring that we can have access to COVID-19 vaccines in our country	3.80	2.58	3.29	2.87
You do not understand why people are reluctant to get vaccinated	3.57	2.56	1.48	2.61
**% of case in each cluster**	**24.9**	**22.8**	**22.4**	**29.9**
	**270**	**248**	**244**	**326**

Source: Eurobarometer 94.3/2021, authors’ analysis. n (valid) = 25,020 (EU27).

## Data Availability

The analyzed Eurobarometer data is available at the following link: https://bit.ly/3xuntiA, accessed on 15 January 2022.

## References

[1] European Commission (2022). Eurobarometer 93.1. https://bit.ly/3mSC3LS.

[2] European Commission (2021). Eurobarometer 94.3. https://bit.ly/3xuntiA.

[3] European Commission (2021). Eurobarometer 95.1. https://bit.ly/3aXCa6d.

[4] Guvernul României Situația Vaccinării în România. https://vaccinare-covid.gov.ro/situatia-vaccinarii-in-romania/.

[5] Cristea D., Ilie D.G., Constantinescu C., Fîrțală V. (2021). Vaccinating against COVID-19: The Correlation between Pro-Vaccination Attitudes and the Belief That Our Peers Want to Get Vaccinated. Vaccines.

[6] Cadeddu C., Sapienza M., Castagna C., Regazzi L., Paladini A., Ricciardi W., Rosano A. (2021). Vaccine Hesitancy and Trust in the Scientific Community in Italy: Comparative Analysis from Two Recent Surveys. Vaccines.

[7] Yaqub O., Castle-Clarke S., Sevdalis N., Chataway J. (2014). Attitudes to Vaccination: A Critical Review. Soc. Sci. Med..

[8] ECDC Vaccine Hesitancy. https://www.ecdc.europa.eu/en/immunisation-vaccines/vaccine-hesitancy.

[9] Elsayed M., El-Abasiri R.A., Dardeer K.T., Kamal M.A., Htay M.N.N., Abler B., Marzo R.R. (2022). Factors Influencing Decision Making Regarding the Acceptance of the COVID-19 Vaccination in Egypt: A Cross-Sectional Study in an Urban, Well-Educated Sample. Vaccines.

[10] Rodrigues F., Block S., Sood S. (2022). What Determines Vaccine Hesitancy: Recommendations from Childhood Vaccine Hesitancy to Address COVID-19 Vaccine Hesitancy. Vaccines.

[11] Sallam M. (2021). COVID-19 vaccine hesitancy worldwide: A concise systematic review of vaccines acceptance rates. Vaccines.

[12] Wagner A.L., Masters N.B., Domek G.J., Mathew J.L., Sun X., Asturias E.J., Ren J., Huang Z., Contreras-Roldan I.L., Gebremeskel B. (2019). Comparisons of vaccine hesitancy across five low- and middle-income countries. Vaccines.

[13] Pomara C., Sessa F., Ciaccio M., Dielim F., Esposito M., Garozzo S.F., Giarratano A., Prati D., Rappa F., Salerno M. (2021). Post-mortem findings in vaccine-induced thrombotic thrombocytopenia. Haematologica.

[14] Lazarus J.V., Ratzan S.C., Palayew A., Gostin L.O., Larson H.J., Rabin K., Kimball S., El-Mohandes A. (2021). A global survey of potential acceptance of a COVID-19 vaccine. Nat. Med..

[15] Nguyen K.H., Srivastav A., Razzaghi H., Williams W., Lindley M.C., Jorgensen C., Abad N., Singleton J.A. (2021). COVID-19 Vaccination Intent, Perceptions, and Reasons for Not Vaccinating Among Groups Prioritized for Early Vaccination—United States, September and December 2020. Am. J. Transplant..

[16] The New York Times (2021). AstraZeneca Concerns Throw Europe’s Vaccine Rollout into Deeper Disarray. https://nyti.ms/3z85xwM.

[17] Time (2021). Why Europe’s AstraZeneca Vaccine Woes Are a Problem for the World. https://bit.ly/3zoAnl1.

[18] European Medicines Agency (2021). AstraZeneca’s COVID-19 Vaccine: EMA Finds Possible Link to Very Rare Cases of Unusual Blood Clots with Low Blood Platelets. https://bit.ly/3wY0UTd.

[19] Likas A., Vlassis N.J., Verbeek J. (2003). The Global K-Means Clustering Algorithm. Pattern Recognit..

[20] European Commission (2021). The EU and the Coronavirus Pandemic. https://data.europa.eu/doi/10.2775/395447.

[21] European Centre for Disease Prevention and Control COVID-19 Vaccine Tracker. https://bit.ly/3z7yhFK.

[22] Cristea D., Ilie D.-G., Constantinescu C., Fîrțală V., Acceptance, Hesitancy, and Refusal in anti-COVID-19 vaccination A Cluster Analysis Aiming at the Typology behind These Three Concepts. BMC Public Health 2022, Under Review. https://bit.ly/39rl7t1.

[23] Vulpe S., Rughiniş C. (2021). Social amplification of risk and “probable vaccine damage”: A typology of vaccination beliefs in 28 European countries. Vaccine.

